# Protective Effects of *Bacopa monnieri* Extract, Mixed Thai Berry Extract and Their Combination Against Chronic Unpredictable Mild Stress-Induced Behavioral Changes in Rats

**DOI:** 10.3390/ph19070981

**Published:** 2026-06-24

**Authors:** Phichsinee Rerkshanandana, Sutisa Nudmamud-Thanoi, Kalyarut Phumlek, Pailada Tiemtad, Prapapan Temkitthawon, Jureepon Roboon, Paweena Kaewman, Wanfrutkon Waehama, Plaiyfah Janthueng, Wiyada Khangkhachit, Sasimontra Timjan, Kornkanok Ingkaninan

**Affiliations:** 1 Department of Pharmaceutical Chemistry and Pharmacognosy, Faculty of Pharmaceutical Sciences and Center of Excellence for Innovation in Chemistry, Naresuan University, Phitsanulok 65000, Thailand; phichsineer65@nu.ac.th (P.R.); kalyarut.ph@nu.ac.th (K.P.); pailadat66@nu.ac.th (P.T.); prapapant@nu.ac.th (P.T.); wiyada.kh@nu.ac.th (W.K.); sasimontra06@gmail.com (S.T.); 2 Research and Innovation Cluster for Natural Health Products, Faculty of Pharmaceutical Sciences, Naresuan University, Phitsanulok 65000, Thailand; 3 Department of Anatomy, Faculty of Medical Science, Naresuan University, Phitsanulok 65000, Thailand; jureeponr@nu.ac.th (J.R.); paweenakae@nu.ac.th (P.K.); wanfrutkonw65@nu.ac.th (W.W.); plaiyfahj66@nu.ac.th (P.J.); 4 Center of Excellence in Medical Biotechnology, Naresuan University, Phitsanulok 65000, Thailand

**Keywords:** *Bacopa monnieri* aerial parts, mixed berry extract, anthocyanins, anxiolytic, recognition memory, Chronic Unpredictable Mild Stress (CUMS)

## Abstract

**Background/Objectives:** Chronic stress contributes to anxiety disorders and cognitive impairment, while effective multi-target therapeutic strategies remain limited. This study investigated the effects of a standardized extract prepared from the aerial parts of *Bacopa monnieri* (L.) Wettst. (Brahmi) extract and an anthocyanin-rich mixed Thai berry extract, administered individually and in combination, in a chronic unpredictable mild stress (CUMS) rat model. **Methods:** Extracts derived from *Morus alba* L. (mulberry), *Antidesma ghaesembilla* Gaertn. (mamao), and *Syzygium nervosum* DC. (ma-kiang) were characterized for anthocyanin and phenolic contents, antioxidant activities, and cyanidin-3-O-glucoside levels using HPLC. Male Sprague Dawley rats were subjected to a 14-day CUMS protocol and treated with Brahmi extract, mixed Thai berry extract, or their combinations. Behavioral assessments included the open-field test, elevated plus maze, and novel object recognition test. Histopathological evaluation of the prefrontal cortex and hippocampus was also performed. **Results:** Brahmi extract and mixed Thai berry extract attenuated selected anxiety-related behaviors and improved recognition memory-related parameters in CUMS-exposed rats. The low-dose berry extract produced the most consistent behavioral improvements, whereas combination-treated groups showed greater histological preservation. Histopathological analysis revealed reduced neuronal degeneration and improved tissue organization in the prefrontal cortex and hippocampus of treated animals. **Conclusions:** These findings support the potential therapeutic relevance of *Bacopa monnieri* and anthocyanin-rich Thai berry extracts under chronic stress conditions, with differential effects observed between individual and combination treatments.

## 1. Introduction

Chronic stress has become a major global health concern, driven by modern lifestyles and increasing psychosocial demands, and is strongly associated with the development of stress-related mental health disorders, including anxiety and cognitive dysfunction [1,2]. Beyond its epidemiological significance, accumulating clinical and experimental evidence indicates that prolonged exposure to chronic stress directly contributes to the development of anxiety disorders and stress-related memory impairment [3,4].

At the neurobiological level, prolonged exposure to stress disrupts neuronal homeostasis in brain regions involved in emotional regulation and cognitive processing, particularly the hippocampus and prefrontal cortex [4,5]. Chronic stress induces oxidative stress and neuroinflammatory responses, leading to neuronal damage and impaired neuroplasticity [5,6,7]. These pathological changes manifest behaviorally as anxiety-like phenotypes and deficits in learning and memory, including impaired recognition memory. Consistent with these findings, animal models of chronic stress exhibit anxiety-like behavior accompanied by cognitive impairment [8]. Accordingly, the chronic unpredictable mild stress (CUMS) model is widely employed as a robust and translational experimental paradigm to investigate stress-induced anxiety and cognitive dysfunction [9,10].

Given the central role of oxidative stress and neuroinflammation in stress-related neuronal dysfunction, there is growing interest in natural products with antioxidant and neuroprotective properties as potential complementary therapeutic strategies. Plant-derived bioactive compounds have demonstrated strong antioxidant and anti-inflammatory activities, supporting neuronal survival and synaptic integrity under pathological conditions [10,11,12]. *Bacopa monnieri* (L.) Wettst. (Plantaginaceae; Brahmi) is a well-established medicinal plant in Ayurvedic medicine, traditionally used for enhancing memory and alleviating anxiety. Previous studies have demonstrated that Brahmi exhibits antioxidant, anti-inflammatory, and neuroprotective activities, which are believed to underlie its beneficial effects on learning and memory [13,14]. Saponin glycosides, particularly jujubogenin and pseudojujubogenin derivatives, have been identified as the principal active constituents contributing to these neuropharmacological effects [14,15]. However, experimental evidence supporting the neuroprotective efficacy of standardized Brahmi extract under chronic stress conditions remains limited, highlighting the need for further investigation.

In parallel, increasing attention has been directed toward anthocyanin-rich berries as natural neuroprotective agents. Anthocyanins, including cyanidin-3-O-glucoside (C3G), possess potent antioxidant and anti-inflammatory properties and have been shown to attenuate oxidative neuronal damage and support cognitive function [11,15,16,17,18]. Several Thai berries, including *Morus alba* L. (Moraceae; mulberry), *Antidesma ghaesembilla* Gaertn. (Phyllanthaceae; mamao), and *Syzygium nervosum* DC. (Myrtaceae; ma-kiang), are rich sources of anthocyanins and phenolic compounds, suggesting their potential to mitigate stress-related neuronal dysfunction through antioxidant mechanisms. Previous studies consistently report strong antioxidant and neuroprotective effects of both saponin-rich Brahmi and anthocyanin-rich berry extracts [14,19,20,21].

Importantly, the use of a mixed-berry formulation offers several potential advantages over single-berry extracts, including broader phytochemical diversity and enhanced antioxidant coverage, which may support multifaceted neuroprotective activity. Combination-based plant formulations may provide a wider range of bioactive constituents than single-plant preparations, as different phenolic compounds, flavonoids, anthocyanins, and other bioactive constituents can act through complementary antioxidant, anti-inflammatory, and neuroprotective pathways. This concept is consistent with previous reports on polyherbal formulations and berry-derived phytochemicals, in which combined plant products have been proposed to exert multi-target biological effects [22,23]. Moreover, combining Brahmi with anthocyanin-rich berry extracts is hypothesized to broaden the spectrum of neuroprotective actions, integrating saponin-mediated neuromodulatory effects with anthocyanin-driven antioxidant and anti-inflammatory properties. To date, however, no study has systematically evaluated the combined effects of standardized Brahmi extract and a standardized anthocyanin-rich mixed Thai berry extract under chronic stress conditions, representing a significant gap in current knowledge and an opportunity to identify novel and unique natural active ingredient combinations with complementary neuroprotective potential.

Based on these considerations, this study aimed to characterize individual Thai berry extracts and the mixed extract through phytochemical profiling, antioxidant assays, and HPLC-based quantification of C3G in the mixed Thai berry extract. The in vivo effects of the standardized anthocyanin-rich mixed Thai berry extract, administered at low and high doses, alone and in combination with Brahmi extract, were then evaluated on anxiety-like behaviors and recognition memory in a CUMS rat model. Histopathological assessment of the prefrontal cortex and hippocampal subregions was also performed to support behavioral outcomes. Together, these findings are expected to advance understanding of herbal neuroprotection and support the development of evidence-based natural therapeutic strategies for stress-related anxiety and cognitive impairments.

## 2. Results

### 2.1. Bioactive Compound Content and Antioxidant Activity Quantification

In the present study, bioactive compound contents and antioxidant activities were quantitatively determined for the Thai berry extracts. Given that the antioxidant and bioactive properties of Brahmi have been extensively documented in previous studies, the present analysis focused on the comparative phytochemical and antioxidant profiling of the Thai berry extracts [19,24]. For consistency and experimental confirmation under identical assay conditions, the antioxidant activity of the Brahmi extract was additionally evaluated. Brahmi exhibited measurable radical scavenging activity, with IC_50_ values of 80.44 ± 4.83 µg/mL in the DPPH assay and 134.27 ± 11.10 µg/mL in the ABTS assay. Trolox, used as a reference antioxidant, showed strong activity in both assays, with IC_50_ values of 3.62 ± 0.92 µg/mL in DPPH and 4.32 ± 0.02 µg/mL in ABTS. These findings are consistent with previously reported antioxidant properties of Brahmi and support its use as a reference phytochemical in the present study.

As summarized in Table 1, TAC varied markedly among the extracts, with mulberry exhibiting the highest TAC, followed by ma-kiang, mixed Thai berry extract, and mamao. The mixed Thai berry extract used in this study consisted of mulberry (*M. alba*), mamao (*A. ghaesembilla*), and ma-kiang (*S. nervosum*) extracts. TPC was highest in ma-kiang, followed by mixed Thai berry extract and mulberry, and was lowest in mamao. Consistent trends were observed across antioxidant assays. In the DPPH assay, ma-kiang demonstrated the strongest radical scavenging activity, followed by mixed Thai berry extract, mulberry, and mamao. While ma-kiang showed the strongest activity in the DPPH assay, the ABTS assay revealed a different pattern, with mulberry exhibiting the lowest IC_50_ value, followed by the mixed Thai berry extract, ma-kiang, and mamao. FRAP values followed the same pattern, with ma-kiang exhibiting the greatest reducing power, followed by mixed Thai berry extract, mulberry, and mamao. These phytochemical and antioxidant profiles may contribute to the differential behavioral and histopathological outcomes observed in the CUMS model, particularly in groups receiving the mixed Thai berry extract.

### 2.2. Quantitative HPLC Analysis of C3G

Quantitative HPLC analysis revealed distinct differences in C3G content among the berry extracts. Ma-kiang contained the highest level of C3G (17.20 ± 0.28 mg/g), followed by mulberry (10.88 ± 0.39 mg/g) and the mixed Thai berry extract (10.44 ± 0.34 mg/g). In contrast, mamao exhibited only a minimal amount (0.21 ± 0.04 mg/g). These findings demonstrate substantial variation in C3G abundance among the berry sources and confirm the suitability of the HPLC method for quantitative anthocyanin determination. Representative HPLC chromatograms of the C3G standard and the standardized anthocyanin-rich mixed Thai berry extract are provided in Figures S1 and S2, respectively, in the Supplementary Materials.

### 2.3. Correlation Between Spectrophotometric and HPLC Analyses of Anthocyanin Content

**Figure 1:**
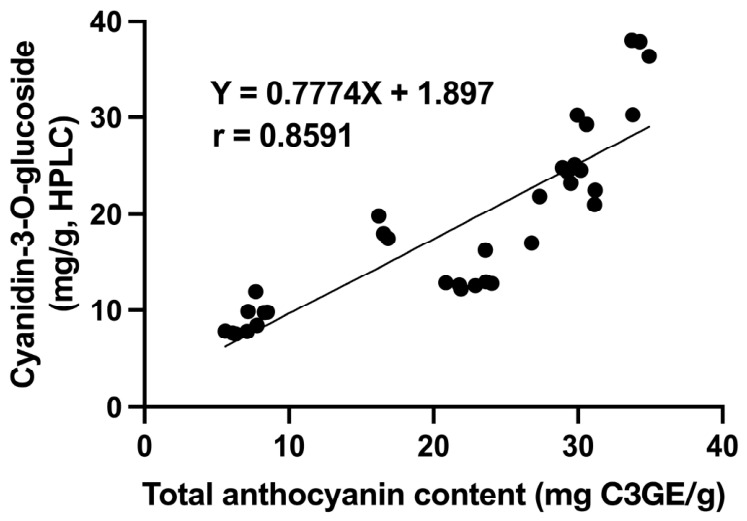
Correlation between spectrophotometric TAC (mg C3GE/g) and HPLC-quantified C3G (mg/g) across berry extracts (*n* = 35), showing a strong positive relationship (r = 0.8591, *p* < 0.0001).

A Pearson correlation analysis was performed between spectrophotometric total anthocyanin content (TAC; mg C3GE/g) and HPLC-derived Cyanidin-3-O-glucoside (C3G; mg/g) to assess the consistency between both analytical methods. Analysis across four berry extracts (mulberry, mamao, ma-kiang, and mixed Thai berry extract; *n* = 35) revealed a strong positive correlation (r = 0.8591, *p* < 0.0001) as shown in Figure 1. Extracts with higher TAC values correspondingly exhibited higher HPLC-quantified C3G levels, indicating that spectrophotometric TAC provides a reliable proxy for relative anthocyanin abundance across these berry extracts.

### 2.4. Behavioral Study

To ensure the reliability and validity of behavioral data, four rats from both the control and chronic unpredictable mild stress (CUMS)-treated groups were excluded from the analysis. Two rats were excluded due to excessive body weight that could potentially affect behavioral performance, and two rats were excluded due to abnormal spontaneous locomotor activity unrelated to the experimental treatments. These exclusions were conducted in accordance with established behavioral phenotyping criteria [25,26,27].

#### 2.4.1. Validation of the CUMS Model

##### Body Weight Changes

**Figure 2:**
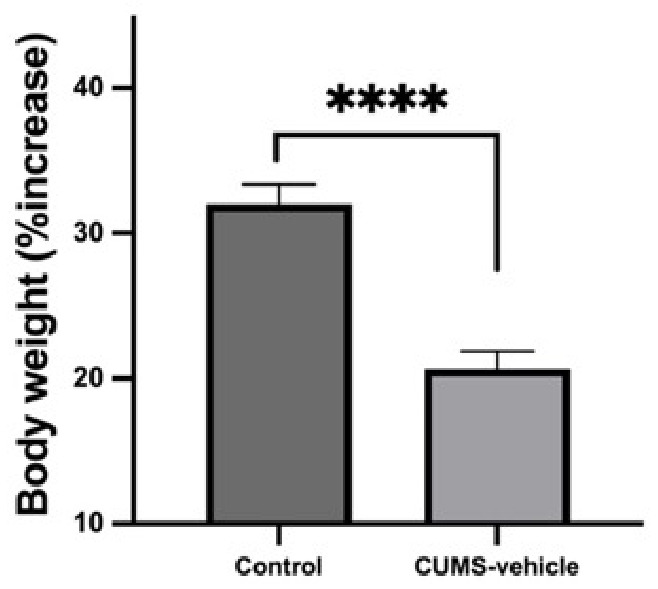
Body weight gain (%increase) after 14 days of experimental treatment. Data are presented as mean ± SEM (*n* = 10–12 per group). Statistical significance was determined by unpaired *t*-test. **** *p* < 0.0001 compared with the control group.

Rats in the CUMS-vehicle group exhibited a significantly lower percentage increase in body weight (20.67 ± 1.21%) compared to the control group (31.99 ± 1.39%) after 14 days (*p* < 0.0001). This reduction in weight gain indicates a physiological consequence of chronic unpredictable mild stress exposure, confirming successful induction of the CUMS model, as shown in Figure 2. Body weight gain data from all experimental groups are additionally provided in Supplementary Figure S3. Although the control group exhibited significantly greater body weight gain than all CUMS-exposed groups, no marked differences were observed among the CUMS-treatment groups during the experimental period.

##### Behavioral Validation of the CUMS Model

After 14 days of CUMS exposure, rats in the CUMS-vehicle group exhibited clear anxiety-like behavioral alterations compared with the control group (Table 2). In the open-field test (OFT), CUMS-vehicle rats showed a substantially greater total distance traveled compared with control rats, although the difference was not statistically significant, suggesting a numerical tendency toward increased locomotor activity. No significant differences were observed in the percentage of time spent in the central and peripheral zones. In the elevated plus maze (EPM), CUMS-vehicle rats traveled longer distances (*p* < 0.01) and entered the closed arms significantly more frequently (*p* < 0.001) than control rats, consistent with increased anxiety-like behavior. In the novel object recognition test (NORT), no significant differences were observed between the control and CUMS-vehicle groups in recognition index, total exploration time, or number of entries to the novel object, indicating that CUMS exposure did not significantly impair recognition memory under the present experimental conditions. Taken together, reduced body weight gain and significant alterations in EPM parameters indicate successful induction of a stress-related anxiety-like phenotype under the present experimental conditions, although recognition memory was not significantly impaired.

#### 2.4.2. Effects of Treatments on Anxiety-Related Behaviors and Recognition Memory in CUMS-Exposed Rats

##### Anxiety-Related Behavioral Assessments

**Figure 3:**
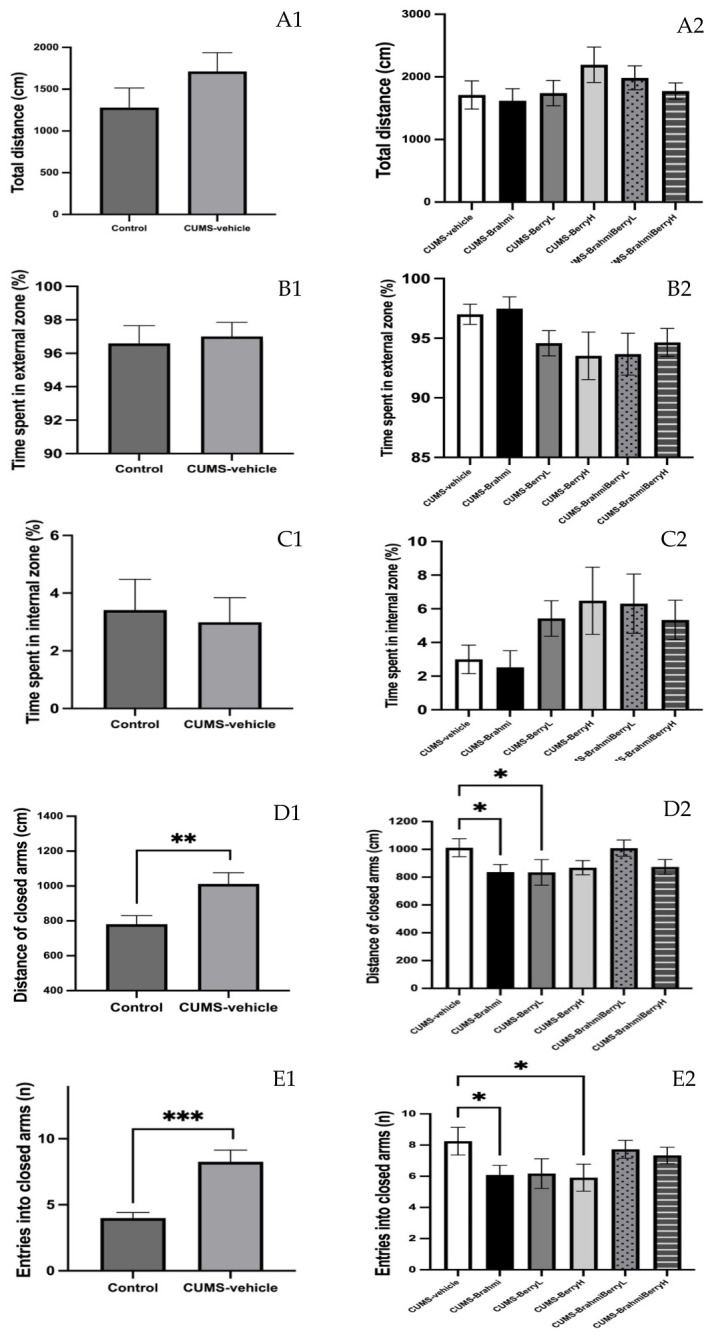
Effects of Brahmi extract, mixed Thai berry extract, and their combination on anxiety-related behaviors in CUMS-exposed rats. Anxiety-related behaviors were evaluated using the open-field test (OFT) and elevated plus maze (EPM). (**A1**) Total distance traveled in the OFT in the control and CUMS-vehicle groups; (**A2**) total distance traveled in the OFT among CUMS-exposed treatment groups; (**B1**) time spent in the external zone of the OFT in the control and CUMS-vehicle groups; (**B2**) time spent in the external zone of the OFT among CUMS-exposed treatment groups; (**C1**) time spent in the internal zone of the OFT in the control and CUMS-vehicle groups; (**C2**) time spent in the internal zone of the OFT among CUMS-exposed treatment groups; (**D1)** distance traveled in the closed arms of the EPM in the control and CUMS-vehicle groups; (**D2**) distance traveled in the closed arms of the EPM among CUMS-exposed treatment groups; and (**E1**) number of closed-arm entries in the EPM in the control and CUMS-vehicle groups; (**E2**) number of closed-arm entries in the EPM among CUMS-exposed treatment groups. Data are presented as mean ± SEM (*n* = 10–12 per group). Comparisons between the control and CUMS-vehicle groups were analyzed using an unpaired *t*-test. Comparisons among CUMS-exposed groups were analyzed using one-way ANOVA followed by Fisher’s LSD post hoc test only when the overall ANOVA was significant. * *p* < 0.05, ** *p* < 0.01, and *** *p* < 0.001 versus the corresponding comparator indicated in the figure. Abbreviations: CUMS, chronic unpredictable mild stress; Brahmi, Brahmi extract-treated group; BerryL, low-dose mixed Thai berry extract-treated group; BerryH, high-dose mixed Thai berry extract-treated group; BrahmiBerryL, Brahmi extract combined with low-dose mixed Thai berry extract-treated group; BrahmiBerryH, Brahmi extract combined with high-dose mixed Thai berry extract-treated group.

All CUMS-induced groups exhibited comparable total distances traveled in the OFT (Figure 3A1,A2), indicating that locomotor activity was not markedly affected by any treatment. Rats treated with BerryL (low-dose mixed Thai berry extract), BerryH (high-dose mixed Thai berry extract), BrahmiBerryL (Brahmi extract combined with low-dose mixed Thai berry extract), and BrahmiBerryH (Brahmi extract combined with high-dose mixed Thai berry extract) spent less time in the external zone (Figure 3B1,B2) and more time in the internal zone (Figure 3C1,C2) compared to the CUMS-vehicle group. Although these differences did not reach statistical significance in the overall one-way ANOVA, exploratory pairwise comparisons using unpaired *t*-tests suggested a consistent trend toward reduced anxiety-like behavior in the treated group. Specifically, for time spent in the external zone, significant or near-significant differences were observed between the CUMS-vehicle and the CUMS-BerryL groups (*p* = 0.0432), with near-significant trends noted for the CUMS-BerryH (*p* = 0.0657), CUMS-BrahmiBerryL (*p* = 0.0541), and CUMS-BrahmiBerryH (*p* = 0.0589) groups. Similarly, for time spent in the internal zone, exploratory analyses revealed a significant increase in the CUMS-BerryL group compared with the CUMS-vehicle group (*p* = 0.0432), while the CUMS-BerryH (*p* = 0.0657), CUMS-BrahmiBerryL (*p* = 0.0557), and CUMS-BrahmiBerryH (*p* = 0.0602) groups showed trends toward increased internal zone exploration.

In the EPM, the distance traveled in the closed arms (Figure 3D1,D2) was significantly reduced in the CUMS-Brahmi (836.30 ± 55.07 cm) and CUMS-BerryL (834.50 ± 91.83 cm) groups compared to the CUMS-vehicle group (1012.00 ± 64.42 cm, *p* < 0.05). Exploratory pairwise comparisons further suggested a reduction in closed-arm distance in the CUMS-BerryH group (*p* = 0.0486), with a near-significant trend also observed in the CUMS-BrahmiBerryH group (*p* = 0.0574). The number of closed-arm entries (Figure. 3E) was significantly higher in the CUMS-vehicle group (8.25 ± 0.89), whereas the CUMS-Brahmi (6.08 ± 0.61) and CUMS-BerryH (5.91 ± 0.87) groups showed significantly fewer entries (*p* < 0.05). Although other treatment groups did not reach statistical significance in the one-way ANOVA, exploratory analysis indicated a trend toward reduced closed-arm entries in the CUMS-BerryL group compared with the CUMS-vehicle group (*p* = 0.0620). The absence of statistically significant overall ANOVA effects in the OFT may reflect the relatively mild anxiety-like phenotype induced by the 14-day CUMS protocol, together with the relatively high variability and lower sensitivity of OFT parameters compared with EPM measurements.

##### Memory and Cognitive Function Evaluations

**Figure 4:**
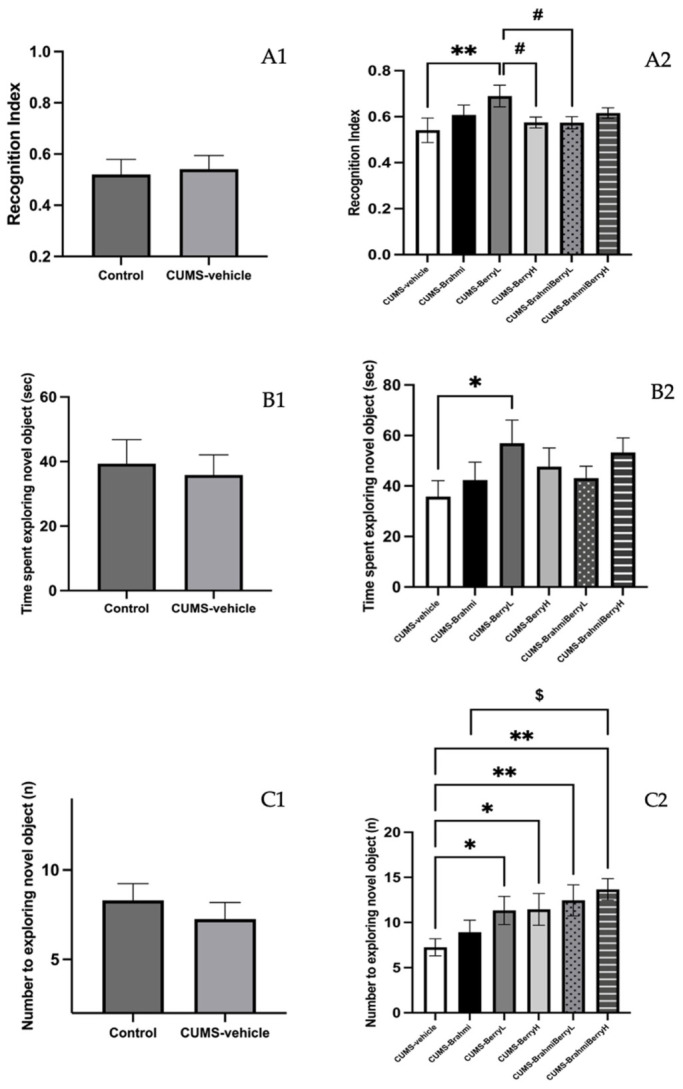
Effects of Brahmi extract, mixed Thai berry extract, and their combination on recognition memory in CUMS-exposed rats. Cognitive performance was assessed using the novel object recognition test (NORT): (**A1**) recognition index in the control and CUMS-vehicle groups; (**A2**) recognition index among CUMS-exposed treatment groups., (**B1**) time spent exploring the novel object in the control and CUMS-vehicle groups; (**B2**) time spent exploring the novel object among CUMS-exposed treatment groups., and (**C1**) number of entries to the novel object in the control and CUMS-vehicle groups; (**C2**) number of entries to the novel object among CUMS-exposed treatment groups. Data are presented as mean ± SEM (*n* = 10–12 per group). Comparisons between the control and CUMS-vehicle groups were analyzed using an unpaired *t*-test. Comparisons among CUMS-exposed groups were analyzed using one-way ANOVA followed by Fisher’s least significant difference (LSD) post hoc test only when the overall ANOVA was significant. * *p* < 0.05 and ** *p* < 0.01 compared with the CUMS-vehicle group; ^#^ *p* < 0.05 compared with the CUMS-BerryL group; ^$^ *p* < 0.05 compared with the CUMS-Brahmi group. Abbreviations: CUMS, chronic unpredictable mild stress; Brahmi, Brahmi extract-treated group; BerryL, low-dose mixed Thai berry extract-treated group; BerryH, high-dose mixed Thai berry extract-treated group; BrahmiBerryL, Brahmi extract combined with low-dose mixed Thai berry extract-treated group; BrahmiBerryH, Brahmi extract combined with high-dose mixed Thai berry extract-treated group.

In the novel object recognition test (NORT), all treatment groups demonstrated higher recognition index values compared with the CUMS-vehicle group (0.54 ± 0.05; Figure 4A1,A2). The CUMS-BerryL group showed a significant increase in recognition index (0.69 ± 0.05) relative to the CUMS-vehicle group (*p* < 0.01) and exhibited significantly greater values than both the CUMS-BerryH and CUMS-BrahmiBerryL groups (*p* < 0.05). The CUMS-BrahmiBerryH group showed a numerically higher recognition index than the CUMS-vehicle group; however, this difference did not reach statistical significance.

Exploration time toward the novel object (Figure 4B1,B2) was increased across all treatment groups with the CUMS-vehicle group (39.31 ± 7.46 s), with the CUMS-BerryL group showing a statistically significant increase (56.96 ± 9.15 s; *p* < 0.05). Other treatment groups showed numerical increases, but these differences were not statistically significant.

For the number of entries to the novel object (Figure 4C1,C2), the CUMS-BerryL (11.33 ± 1.55) and CUMS-BerryH (11.45 ± 1.76) groups exhibited significantly higher values com-pared with the CUMS-vehicle group (7.25 ± 0.94; *p* < 0.05). The CUMS-BrahmiBerryL (12.45 ± 1.72) and CUMS-BrahmiBerryH (13.67 ± 1.19) groups showed an even more pronounced increase (*p* < 0.01), with the CUMS-BrahmiBerryH group exhibiting significantly more novel-object entries than the CUMS-Brahmi group (*p* < 0.05). Overall, the NORT findings demonstrated treatment-related improvements in recognition memory-associated parameters across several groups. The low-dose berry extract showed the most consistent statistically significant effects in selected parameters, whereas the combination-treated groups also demonstrated notable improvements, particularly in novel-object exploration behavior.

### 2.5. Histopathological Analysis of Brain Regions

Histopathological evaluation was performed qualitatively based on descriptive examination of neuronal morphology observed in hematoxylin and eosin (H&E)-stained sections.

#### 2.5.1. Prefrontal Cortex

**Figure 5:**
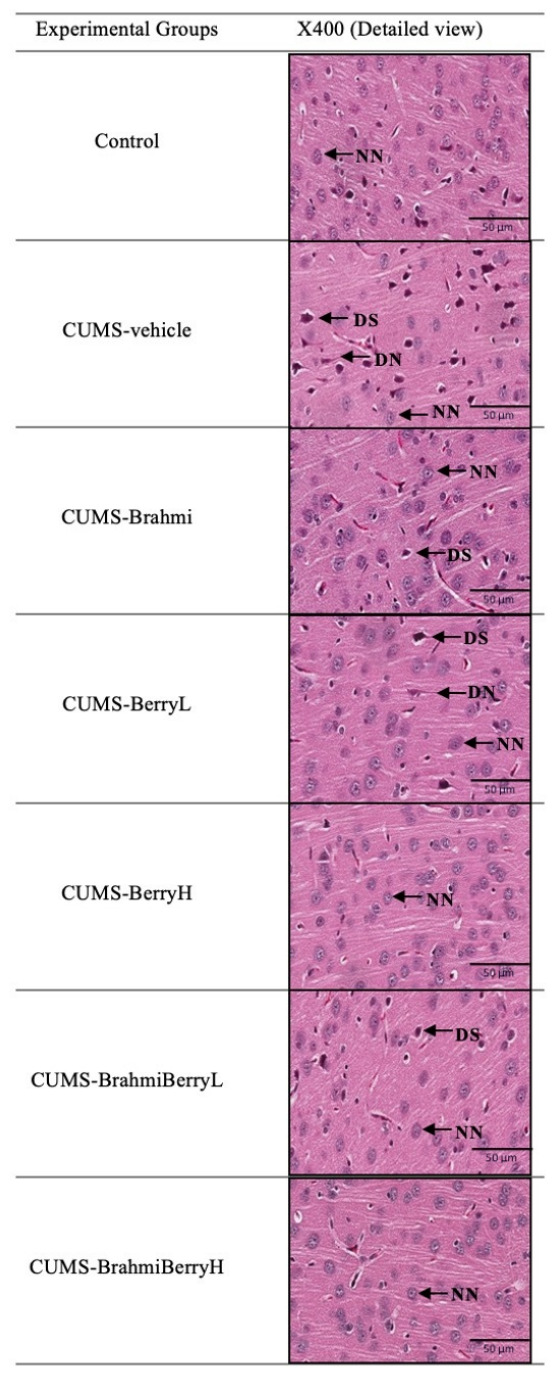
Representative H&E-stained sections of the prefrontal cortex at ×400 magnification. (NN, normal neuron; DS, dark shrunken neuron; DN, dark neuron). Abbreviations: CUMS, chronic unpredictable mild stress; Brahmi, Brahmi extract-treated group; BerryL, low-dose mixed Thai berry extract-treated group; BerryH, high-dose mixed Thai berry extract-treated group; BrahmiBerryL, Brahmi extract combined with low-dose mixed Thai berry extract-treated group; BrahmiBerryH, Brahmi extract combined with high-dose mixed Thai berry extract-treated group.

H&E-stained sections of the prefrontal cortex revealed distinct qualitative differences in neuronal morphology among experimental groups (Figure 5). The CUMS-vehicle group exhibited the most pronounced histopathological alterations, with frequent observation of dark shrunken neurons with reduced cell volume and hyperchromatic cytoplasm and dark neurons with condensed, intensely stained soma, indicating marked stress-associated neuronal damage.

In the CUMS-BerryL group, partial histological improvement was observed; however, dark shrunken neurons and dark neurons were still commonly detected, suggesting limited attenuation of cortical neuronal injury at the low berry dose. The CUMS-Brahmi group demonstrated moderate preservation of neuronal morphology, characterized by a greater presence of normal neurons with intact cell bodies and lightly stained nuclei and fewer degenerative features compared with the CUMS-vehicle group.

Further improvement was evident in the CUMS-BrahmiBerryL group, where cortical architecture appeared largely preserved, with normal neurons predominating and only occasional dark shrunken neurons observed. Marked neuronal preservation was also evident in the CUMS-BerryH group, in which neuronal morphology was predominantly classified as normal neurons, with minimal degenerative features observed. The greatest degree of neuronal preservation was observed in the CUMS-BrahmiBerryH group, whose cortical morphology closely resembled that of the control group, with neurons predominantly classified as normal neurons.

Given the close functional association between the prefrontal cortex and hippocampus in stress regulation and cognitive processing, histopathological alterations in the hippocampus were subsequently examined.

#### 2.5.2. Hippocampus

**Figure 6:**
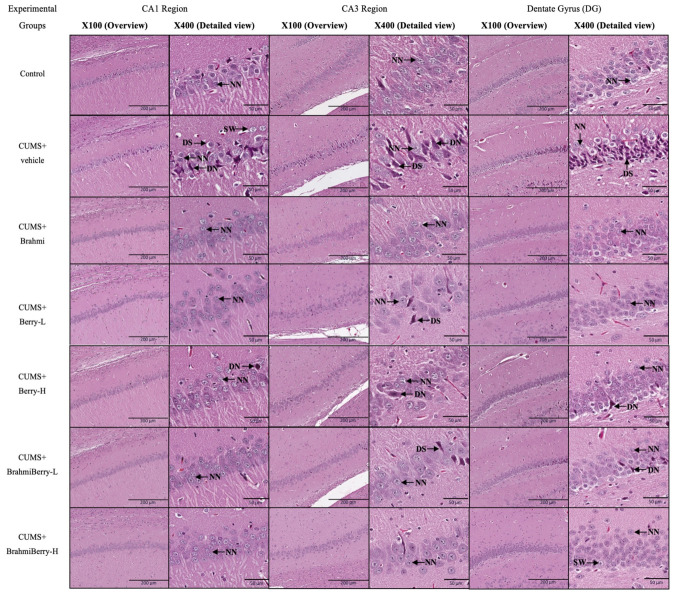
Representative histological sections of hippocampal subregions (CA1, CA3, and DG) stained with H&E at ×100 and ×400 magnification. (NN, normal neuron; DS, dark shrunken neuron; DN, dark neuron; SW, swollen neuron). Abbreviations: CUMS, chronic unpredictable mild stress; Brahmi, Brahmi extract-treated group; BerryL, low-dose mixed Thai berry extract-treated group; BerryH, high-dose mixed Thai berry extract-treated group; BrahmiBerryL, Brahmi extract combined with low-dose mixed Thai berry extract-treated group; BrahmiBerryH, Brahmi extract combined with high-dose mixed Thai berry extract-treated group.

Qualitative histopathological examination of the hippocampus, including the CA1, CA3, and dentate gyrus (DG) subregions, revealed distinct patterns of neuronal morphology among experimental groups (Figure 6).

The CUMS-vehicle group exhibited the most pronounced hippocampal alterations. In the CA1 region, neurons displayed a heterogeneous morphology, including dark shrunken neurons, dark neurons, swollen neurons, and normal neurons. The CA3 region showed frequent dark shrunken neurons and dark neurons, accompanied by some normal neurons, while the DG predominantly exhibited dark shrunken neurons with a limited presence of normal neurons, indicating widespread stress-associated neuronal injury across hippocampal subregions. In the CUMS-BerryH group, neuronal degeneration remained evident but was less extensive. Both CA1 and CA3 contained dark neurons together with normal neurons, whereas the DG similarly showed a mixed population of dark neurons and normal neurons, suggesting partial structural preservation. The CUMS-BrahmiBerryL group demonstrated further improvement in hippocampal morphology. In CA1, neurons were predominantly normal neurons. The CA3 region exhibited occasional dark shrunken neurons alongside normal neurons, while the DG showed both dark neurons and normal neurons, indicating moderate attenuation of stress-induced neuronal alterations. Greater neuronal preservation was evident in the CUMS-BerryL group. Neurons in CA1 were predominantly normal neurons, with the CA3 region showing limited dark shrunken neurons alongside normal neurons and the DG displaying exclusively normal neurons, reflecting improved structural integrity across hippocampal subregions. In the CUMS-BrahmiBerryH group, hippocampal architecture was largely preserved. Neurons in both CA1 and CA3 were classified as normal neurons, while the DG showed predominantly normal neurons with occasional swollen neurons, suggesting residual cellular stress despite overall morphological preservation. The CUMS-Brahmi group exhibited marked preservation of hippocampal neuronal morphology across CA1, CA3, and DG, with neurons consistently appearing as normal neurons, closely resembling the normal structural pattern.

Similarly, the control group displayed intact hippocampal architecture, characterized by well-organized neuronal layers in CA1 and CA3 and a preserved DG, with neurons uniformly appearing as normal neurons.

## 3. Discussion

The present study integrated phytochemical characterization with behavioral and histopathological analyses to evaluate the neuroprotective effects of Brahmi and anthocyanin-rich Thai berry extracts, administered both individually and in combination, in a CUMS rat model. The antioxidant and bioactive properties of Brahmi have been well documented in previous studies, in which phenolic compounds and saponin-rich fractions were shown to exhibit significant radical scavenging and reducing activities across multiple in vitro assays [19,24]. Accordingly, this analysis focused on comparative profiling of Thai berry extracts, with a standardized Brahmi extract employed as a reference intervention. The present findings do not allow confirmation of pharmacological synergy. Rather, they suggest that the combined formulation may differentially affect structural and behavioral endpoints.

Phytochemical analyses demonstrated distinct differences among berry extracts, with ma-kiang exhibiting the highest antioxidant activity, corresponding to its elevated phenolic and anthocyanin contents. Mulberry and the mixed Thai berry extract exhibited moderate antioxidant capacities, whereas mamao showed lower activity. These findings are consistent with previous reports identifying phenolic compounds and anthocyanins as major contributors to antioxidant potential [10,11,12]. Accordingly, the inclusion of ma-kiang and mulberry in the mixed Thai berry extract is supported by their strong phytochemical profiles, while mamao may contribute complementary phytochemical diversity and favorable organoleptic properties. HPLC analysis confirmed C3G as a predominant anthocyanin in ma-kiang and mulberry extracts, with intermediate levels detected in the mixed-berry extract. The strong correlation between spectrophotometric TAC and HPLC-derived C3G (r = 0.8591, *p* < 0.0001) supports the reliability of the pH differential method as a rapid screening tool, while HPLC remains essential for precise quantification of individual anthocyanins. Minor deviations between methods may reflect the contribution of additional anthocyanin species beyond C3G. The stronger antioxidant capacity of ma-kiang and the mixed Thai berry extract supports their potential relevance in modulating stress-associated neurobehavioral alterations. However, the behavioral outcomes did not strictly follow the in vitro antioxidant ranking, suggesting that in vivo efficacy may also depend on bioavailability, metabolism, dose–response characteristics, and interactions among phytochemicals. This observation highlights the complexity of in vivo neuroprotective responses, in which behavioral outcomes may be influenced not only by antioxidant capacity but also by pharmacokinetic properties and interactions among phytochemical constituents.

Importantly, in vivo efficacy was evaluated using a standardized anthocyanin-rich mixed Thai berry extract, rather than individual berry extracts, to reflect the use of multi-component herbal preparations and potential phytochemical complementarity. Although individual berries such as ma-kiang and mulberry exhibited strong antioxidant activity in vitro, the mixed Thai berry extract integrates anthocyanins and phenolic compounds from the three berries, which may provide broader antioxidant coverage under in vivo conditions.

In vivo, the CUMS model was successfully validated by reduced body weight gain and increased anxiety-like behaviors, including elevated closed-arm activity in the EPM and altered exploratory patterns in the OFT, consistent with previous reports linking chronic stress to metabolic and behavioral dysregulation [28,29]. Previous studies have demonstrated that the behavioral and cognitive consequences of CUMS are highly dependent on stress duration, stress severity, and experimental conditions. Although recognition memory impairment has frequently been reported following prolonged CUMS exposure, cognitive outcomes remain variable across different CUMS protocols and experimental settings [30,31].

Accordingly, comparisons between the control and CUMS-vehicle groups were primarily used for model validation, whereas subsequent analyses among CUMS-exposed groups were intended to evaluate treatment-related cognitive modulation under chronic stress conditions.

Therefore, under the present experimental conditions, the NORT paradigm may primarily reflect sensitivity to treatment-related modulation rather than reversal of established cognitive impairment. Consequently, the observed changes in recognition memory-associated parameters should be interpreted as cognitive-enhancing or modulatory effects, rather than restoration of impaired cognitive function. Regarding anxiety-related behaviors, treatment with Brahmi or Thai berry extracts alone produced measurable anxiolytic-like effects in CUMS-exposed rats. Both interventions reduced indices of anxiety in the OFT and EPM, such as closed-arm activity and exploratory avoidance, consistent with the reported neuroprotective and cognitive-enhancing effects of Brahmi, which have been linked to its antioxidant and anti-inflammatory actions [14], and the antioxidant and anti-inflammatory actions of anthocyanins [11,15,18,32]. In contrast, combined Brahmi–berry treatments did not consistently enhance anxiolytic outcomes beyond those observed with individual extracts. This absence of additive benefit in anxiety-related measures may reflect suboptimal dose ratios under the current experimental conditions. Additionally, the relatively short CUMS duration may have constrained the detection of more robust synergistic effects on anxiety-like behavior.

In contrast to anxiety outcomes, cognitive performance assessed by the NORT revealed a distinct response pattern. Berry extracts administered alone modulated recognition memory-associated parameters, including recognition index, exploration time, and novel-object entries. The low-dose berry extract consistently demonstrated the strongest effects across several cognitive parameters, suggesting a potential non-linear or hormetic dose–response relationship, particularly with respect to recognition memory-related parameters in the low-dose berry-treated group compared with the CUMS-vehicle group. These behavioral outcomes were not directly proportional to the in vitro antioxidant ranking of the extracts, suggesting that additional factors such as phytochemical interactions, bioavailability, or neurobiological pathway modulation may contribute to the observed in vivo effects. Furthermore, the differential responses observed between single and combination treatments may indicate that combined phytochemical formulations do not necessarily produce additive behavioral effects under the present experimental conditions. In contrast, combination treatments did not uniformly outperform single treatments in behavioral outcomes, although they demonstrated more pronounced histological preservation. This discrepancy between structural and behavioral findings may reflect differential sensitivity of endpoints or suboptimal dose ratios in the combined formulation. These effects may reflect the combined influence of Brahmi-associated neuromodulatory and synaptic plasticity-supporting properties [14,33], together with the antioxidant and anti-inflammatory actions of berry anthocyanins [11,15,32]. Brahmi has been extensively investigated for its neuroprotective and cognition-enhancing properties, which are largely attributed to saponin glycosides. Previous studies have shown that those saponin glycosides may exert antioxidant and anti-inflammatory effects, support synaptic plasticity, and protect neurons against stress-associated damage. These reported pharmacological properties may have contributed, at least in part, to the behavioral and histopathological effects observed in the present study.

The histopathological observations were consistent with the behavioral outcomes and provided qualitative structural support for the observed functional changes. In the prefrontal cortex, CUMS exposure was associated with clear neuronal degeneration, characterized by the presence of dark shrunken and dark neurons, whereas treatment with Brahmi or Thai berry extracts alone attenuated these morphological alterations to varying degrees. Combination treatments, particularly at the higher dose, showed the greatest preservation of cortical neuronal morphology. However, this structural protection did not translate into proportionally greater anxiolytic effects, suggesting that cortical morphological integrity alone may not fully account for anxiety-related behavioral outcomes under the present experimental conditions.

In contrast, histopathological changes in the hippocampus exhibited a clearer treatment-dependent pattern. Progressive restoration of neuronal morphology across hippocampal subregions (CA1, CA3, and dentate gyrus) was observed among treatment groups, with marked preservation in the Brahmi and combination-treated groups. This regional hippocampal preservation, particularly evident in the combination-treated groups, closely paralleled improvements in recognition memory observed in the novel object recognition test, supporting the well-established association between hippocampal neuronal integrity and cognitive performance under chronic stress conditions [34].

The neuroprotective effects observed in this study are likely mediated, at least in part, by mechanisms previously reported for both Brahmi constituents and anthocyanins. Saponin glycosides, including bacoside A3, bacopaside X, bacopaside I, bacopaside II, and bacopasaponin C, are among the principal bioactive constituents of Brahmi and have been reported to possess antioxidant, anti-inflammatory, neuroprotective, and neurotransmitter-modulating activities that may contribute to stress resilience and cognitive function [35,36]. At the molecular level, previous studies have suggested that standardized Brahmi extracts may influence serotonergic and cholinergic neurotransmission, including modulation of 5-HT-related signaling, acetylcholine-associated pathways, and downstream molecular events linked to synaptic plasticity and memory formation, such as ERK/CREB signaling and synaptic protein regulation [36]. Similarly, anthocyanins have been reported to exert neuroprotective effects through modulation of oxidative stress, neuroinflammatory pathways, and neurobiological processes associated with stress-related behaviors and cognitive performance [37,38]. These reported biological activities may provide a plausible explanation for the behavioral and histopathological effects observed in the present study. However, because direct biochemical, molecular, and neurotransmitter-related measurements were not performed, the proposed mechanisms remain speculative and require confirmation in future studies incorporating appropriate biochemical endpoints.

Collectively, these findings indicate that while Brahmi and Thai berry extracts individually exert anxiolytic effects under chronic stress, their combination does not further enhance anxiety-related behaviors within the present experimental framework. In contrast, cognitive-related outcomes were improved mainly by single-extract treatments, particularly the low-dose berry extract, whereas combination treatments showed stronger histological preservation than behavioral superiority. These findings reveal a differential response between behavioral and structural outcomes, underscoring the multifaceted nature of the observed neuroprotective effects. Importantly, this study introduces a novel framework integrating phytochemical standardization with functional neuroprotection in a clinically relevant stress model.

## 4. Materials and Methods

### 4.1. Chemical and Reagents

Reagents for antioxidant assays, including the Folin–Ciocalteu reagent, 2,2-diphenyl-1-picrylhydrazyl (DPPH), gallic acid, Trolox (6-hydroxy-2,5,7,8-tetramethylchroman-2-carboxylic acid), ABTS (2,2′-azino-bis(3-ethylbenzothiazoline-6-sulfonic acid)), and TPTZ (2,4,6-tri(2-pyridyl)-s-triazine), were purchased from Sigma-Aldrich (St. Louis, MO, USA). Ethanol (95%, food grade) and acetic acid (99%, food grade) were used for extraction. C3G (HPLC standard, >95% purity) was obtained from Glentham Life Sciences Ltd. (Corsham, UK). Acetonitrile (HPLC grade) was supplied by Fisher Scientific (Seoul, South Korea), and formic acid (99%, analytical grade) was purchased from CARLO ERBA Reagents (Cornaredo, Italy). Ultrapure water (18 MΩ·cm) was produced using a Milli-Q^®^ Reference Water Purification System (Merck Millipore, Burlington, MA, USA). Reference standards for bacoside A3, bacopaside X, bacopaside I, bacopaside II, and bacopasaponin C were obtained from MedChemExpress (Monmouth Junction, NJ, USA) with purity ≥98.0%.

### 4.2. Plant Materials

Aerial parts of Brahmi were collected from Phitsanulok Province, Thailand. The plant material was authenticated, and a voucher specimen (Phrompittayarat001) was deposited at the Herbarium of Mahidol University (PBM), Thailand. The collected samples were thoroughly cleaned and dried in a hot-air oven at 50 °C for 24 h, then ground and sieved through a 60-mesh sieve. The resulting powder was stored at −20 °C until further phytochemical analysis and extraction, following previously established protocols [39,40].

Ripe fruits of *Morus alba* L. or mulberry (Chiang Mai variety, voucher specimen PNU 6078) were obtained from the Queen Sirikit Sericulture Center, Nan Province, Thailand. *Antidesma ghaesembilla* Gaertn. (Fa Prathan variety, voucher specimen PNU 6079) or mamao fruits were collected from the Sakon Nakhon Markmao Association, Sakon Nakhon Province, Thailand, and *Syzygium nervosum* DC. or ma-kiang fruits (voucher specimen PNU 6080) were sourced from Maejo University, Phrae Campus, Thailand. All voucher specimens were deposited at the Herbarium, Biology Department, Faculty of Science, Naresuan University, Thailand. All fruit samples were harvested at the fully ripe stage and stored at −20 °C prior to extraction.

### 4.3. Extract Preparation

The extraction methods were selected based on a review of previous studies, considering active compound concentration, safety, and cost-effectiveness. The Brahmi extract was prepared following established protocols [40], with specific steps modified to improve the extraction efficiency and overall extract quality. The Brahmi extract was standardized to contain 5.0% (*w*/*w*) total saponin glycosides, as determined by an HPLC method (see Section 4.4.1). The extraction yield of Brahmi extract was 10–13% based on dried plant material.

Thai berries were extracted with 5% acetic acid in 95% ethanol as previously described [41], with minor modifications based on established methods for anthocyanin-rich extracts [42]. The extraction yields were 9.19% for mulberry, 15.89% for mamao, and 4.40% for ma-kiang, calculated on a fresh weight basis.

The standardized anthocyanin-rich mixed Thai berry extract was prepared by combining extracts of mulberry, mamao, and ma-kiang using an optimized formulation approach designed to enhance anthocyanin content and organoleptic properties, as previously described [41]. The formulation was standardized based on total anthocyanin content (TAC), which served as the reference parameter for dose normalization in subsequent in vivo studies. The standardized anthocyanin-rich mixed Thai berry extract showed a TAC of 41.70 ± 1.66 mg C3G equivalents (C3GE)/g extract, determined using the pH differential method.

For clarity, the standardized anthocyanin-rich mixed Thai berry extract is hereafter referred to as the mixed Thai berry extract.

### 4.4. Quantification of Bioactive Compounds

#### 4.4.1. HPLC Determination of Total Saponin Glycosides in Brahmi

Total saponin glycosides in the Brahmi extract were quantified by HPLC according to a previously validated method [39,40]. The analysis was based on the combined determination of jujubogenin-type glycosides (bacoside A3 and bacopaside X) and pseudojujubogenin-type glycosides (bacopaside I, bacopaside II, and bacopasaponin C). The total saponin content was calculated as the sum of these marker compounds and expressed as % (*w*/*w*) of dried extract.

#### 4.4.2. Determination of Total Anthocyanin Content (TAC)

TAC was quantified using the pH differential method. Extracts (250 µL) were mixed with 750 µL of either 0.025 M potassium chloride buffer (pH 1.0) or 0.04 M sodium acetate buffer (pH 4.5) and incubated in the dark for 15 min, followed by centrifugation at 2500× *g* for 10 min. Absorbance was measured at 510 and 700 nm. Results were expressed as mg C3G equivalents per gram of extract (mg C3GE/g), calculated as previously described [42,43].

#### 4.4.3. Determination of Total Phenolic Content (TPC)

Total phenolic content was determined using the Folin–Ciocalteu method in 96-well microplates [44]. Briefly, 25 µL of the sample was mixed with 25 µL of diluted Folin–Ciocalteu reagent and 200 µL of water. After 5 min, 25 µL of 10.6% sodium carbonate was added, and the mixture was incubated in the dark at room temperature for 60 min. Absorbance was measured at 725 nm. Results were expressed as mg gallic acid equivalents per gram of sample (mg GAE/g).

#### 4.4.4. HPLC Quantification of C3G

C3G was quantified by HPLC using on an Agilent 1260 Infinity system with detection set at 520 nm. Chromatographic separation was achieved on a Phenomenex C12 column (250 × 4.6 mm, 4 µm) equipped with a guard column, using a gradient of solvent A (water: acetonitrile: formic acid, 87:3:10 *v*/*v*/*v*) and solvent B (water: acetonitrile: formic acid, 40:50:10 *v*/*v*/*v*). The flow rate was 1.0 mL/min, the injection volume was 20 µL, and the column temperature was 30 °C. Sample preparation and chromatographic conditions were adapted from previous reports [45], with minor modifications to improve analytical efficiency. The calibration curve showed good linearity over the range of 1.25–40 µg/mL, where R^2^ = 0.993 LOD and LOQ were 0.039 and 1.25 µg/mL, respectively. Representative HPLC chromatograms of standardized C3G and the anthocyanin-rich mixed Thai berry extract are provided in Supplementary Materials: Figures S1 and S2.

### 4.5. Correlation Analysis Between Spectrophotometric and HPLC Anthocyanin Data

To evaluate the consistency between analytical methods for anthocyanin determination, Pearson’s correlation was performed between TAC measured by the pH differential spectrophotometric method and C3G quantified by HPLC. TAC values were plotted against corresponding C3G concentrations, and correlation coefficients (r) with *p*-values were calculated using GraphPad Prism (version 10).

### 4.6. Evaluation of Antioxidant Potential

The antioxidant activities of individual berry extracts and the mixed Thai berry extract were evaluated using DPPH, ABTS, and FRAP assays according to previously established protocols [32,44,46], with minor modifications as described below.

#### 4.6.1. DPPH Radical Scavenging Method

The antioxidant activity of the extracts was evaluated using a modified DPPH radical scavenging assay [44]. Briefly, 150 µL of 0.2 mM DPPH solution was mixed with 75 µL of diluted extract, with a reagent-only control prepared simultaneously. After incubation in the dark at room temperature for 30 min, absorbance was measured at 515 nm using a microplate reader. IC_50_ values were calculated via nonlinear regression using GraphPad Prism (version 10), with Trolox serving as the positive control. Percentage inhibition was determined according to established protocols.

#### 4.6.2. ABTS Radical Scavenging Method

ABTS radical scavenging activity was measured using a modified method adapted from previous studies [32,46]. The ABTS•^+^ reagent was generated by reacting 7 mM ABTS with 2.45 mM potassium persulfate and incubating in the dark at room temperature for 16 h; then, it was diluted 1:24 (*v*/*v*) with water. Extract samples (20 µL) were mixed with 180 µL of diluted ABTS solution and incubated for 6 min at room temperature. Absorbance was recorded at 732 nm using a microplate reader. Trolox served as the positive control and IC_50_ values were calculated via nonlinear regression using GraphPad Prism (version 10). Percentage inhibition was determined according to established protocols.

#### 4.6.3. Ferric Reducing Antioxidant Power (FRAP)Method

The ferric reducing ability of berry extracts was assessed using a modified FRAP assay based on previous studies [32,46], Briefly, 20 µL of sample was mixed with 180 µL of FRAP reagent, prepared by combining acetate buffer (300 mM, pH 3.6), TPTZ solution (10 mM), and FeCl_3_ solution (20 mM) in a 10:1:1 ratio. The mixture was incubated in the dark at room temperature for 30 min, then absorbance was measured at 595 nm using a microplate reader. A standard curve was constructed using FeSO_4_·7H_2_O, and results were expressed as grams of Fe^2+^ equivalents per 100 g of sample. Trolox was used as a positive C.

### 4.7. In Vivo Study

#### 4.7.1. Ethical Approval and Animal Care

The study protocol was approved by the Naresuan University Animal Care and Use Committee (NUACUC; approval no. NU-AE660305). A total of 84 male Sprague Dawley rats (7 weeks old) were obtained from Nomura Siam International Co., Ltd. All animals were housed at Naresuan University under controlled conditions (22 ± 1 °C, 12/12 h light–dark cycle, 55 ± 10% humidity) with ad libitum access to food and water. Animals were allowed a 7-day acclimatization period before the experiment, after which they were randomly assigned to either the chronic unpredictable mild stress (CUMS) group or the control group. All experimental procedures were conducted in compliance with relevant laws and institutional guidelines.

#### 4.7.2. The Chronic Unpredictable Mild Stress Procedure

Chronic unpredictable mild stress (CUMS) is a validated rodent model for studying stress-related disorders, characterized by the induction of depressive- and anxiety-like behaviors through repeated exposure to mild, unpredictable stressors. It mimics the chronic and variable nature of human psychological stress and is widely used to evaluate therapeutic interventions [47,48]. Animals were exposed to various mild stressors in an unpredictable sequence, as detailed in Table 3.

#### 4.7.3. Administration of Treatment

A total of 84 male Sprague Dawley rats were randomly allocated into seven groups (*n* = 12 per group). The normal control group (control) received reverse osmosis (RO) water (10 mL/kg bw, p.o.). The remaining six groups were subjected to the CUMS protocol. Among them, the CUMS control group (CUMS-vehicle) also received RO water (10 mL/kg bw, p.o.). The treatment groups consisted of the CUMS-Brahmi group receiving Brahmi extract at 20 mg/kg bw; the CUMS-BerryL and CUMS-BerryH groups, which received low and high doses of mixed Thai berry extract, corresponding to 0.043 and 0.215 mg C3GE/kg bw, respectively; and the CUMS-BrahmiBerryL and CUMS-BrahmiBerryH groups, which received Brahmi extract in combination with low and high doses of mixed Thai berry extract, respectively. All treatments were administered orally once daily in the morning for 14 consecutive days. The dose of Brahmi extract (20 mg/kg bw) was selected based on previous studies reporting cognitive-enhancing and neuroprotective effects in rodent models [33,49]. Dose selection for the mixed Thai berry extract was guided by reports on anthocyanin-rich berries demonstrating cognitive benefits [50]. For the combination groups, Brahmi extract was administered at 20 mg/kg bw together with the mixed Thai berry extract at either 0.043 or 0.215 mg C3GE/kg bw. The combination was freshly prepared before administration by mixing the corresponding doses of each standardized extract in RO water to achieve the same final administration volume.

#### 4.7.4. Body Weight Monitoring

All animals were weighed each morning before dosing for 14 consecutive days using a calibrated digital balance. Body weight (BW) data were used to calculate the percent increase from Day 0 to Day 14.

%Increase = [(BW14 − BW0)/BW0] × 100,

where BW0 denotes the baseline (Day 0) body weight and BW14 the Day 14 body weight; positive values indicate gain relative to the baseline, negative values indicate loss, and 0% indicates no change.

#### 4.7.5. Behavioral Assessments

##### Open-Field Test (OFT)

The open-field test (OFT) is a well-established behavioral paradigm used to assess spontaneous locomotor activity and general arousal in rodents. It also provides an indirect measure of anxiety-like behavior based on the animal’s tendency to avoid open and unprotected areas of the arena [28]. In the present study, each rat was placed individually at the center of the open-field arena and was allowed to explore freely for 10 min. The arena was divided into predefined zones, and locomotor activity and movement patterns during the last 5 min of the test were recorded for analysis.

##### Elevated Plus Maze Test (EPM)

The elevated plus maze (EPM) is a standard behavioral test for assessing anxiety-related responses in rodents. It consists of two open arms and two closed arms elevated above the floor. Rodents naturally avoid open arms, and the time spent and entries into the open arms serve as indices of anxiety-like behavior [29]. In the present study, each rat was placed at the center of the maze and allowed to explore freely for 10 min. The time spent and number of entries into the open and closed arms during the last 5 min of the test were recorded for the evaluation of anxiety-like behavior.

##### Novel Object Recognition Test (NOR)

The novel object recognition (NOR) test is a widely used behavioral paradigm for assessing recognition memory in rodents. The test is based on the natural tendency of rodents to preferentially explore a novel object over a familiar one. Increased exploration time of the novel object reflects intact recognition memory [51,52]. In the present study, each rat was placed individually in the arena during the training phase and was allowed to explore two identical objects. After a retention interval of 90 min, the rat was returned to the arena for the test phase, during which one familiar object was replaced with a novel object. The time spent exploring the novel object and the number of entries into the novel object zone were recorded for the evaluation of recognition memory.

Behavioral recordings from the OFT, EPM, and NORT were analyzed using SMART video tracking software V3.0 (Panlab, Spain). To improve data reliability and minimize observer bias, behavioral parameters were independently verified by three investigators through repeated assessments. The final datasets were confirmed based on concordant evaluations obtained from both automated software analysis and investigator-based observations.

### 4.8. Histological Analyses

After the behavioral tests, rats were anesthetized with thiopental sodium (100 mg/kg, intraperitoneally). The prefrontal cortex and hippocampus tissues from six selected rats in each group were dissected and fixed in 10% neutral buffered formalin. The samples were then processed using a standard paraffin-embedding protocol, sectioned at approximately 5 μm thickness using a rotary microtome, and stained with hematoxylin and eosin (H&E). Finally, the sections were mounted on glass slides and examined under a bright-field light microscope (Olympus BX51, Olympus, Tokyo, Japan) to evaluate histopathological changes [53].

### 4.9. Statistical Analysis

Data were presented as mean ± standard deviation and the difference between variables was analyzed using the independent sample *t*-test or Mann–Whitney test (non-parametric). A *p* < 0.05 was considered statistically significant.

All data were analyzed using GraphPad Prism version 10. Data are presented as mean ± SEM unless otherwise indicated. Comparisons between two groups were analyzed using an unpaired *t*-test. Comparisons among three or more groups were analyzed using one-way ANOVA followed by Fisher’s LSD post hoc test only when the overall ANOVA was significant. Statistical significance was set at *p* < 0.05.

## 5. Conclusions

This study integrated phytochemical characterization with in vivo behavioral and histopathological analyses to evaluate the neuroprotective effects of standardized Brahmi, anthocyanin-rich Thai berry extracts, and their combination in a chronic unpredictable mild stress (CUMS) rat model. Thai berry extracts exhibited high anthocyanin with strong antioxidant capacity, and HPLC analysis confirmed C3G as a major anthocyanin, supporting rapid screening approaches for anthocyanin-rich extracts.

Behaviorally, CUMS reliably induced anxiety-like phenotypes in rats. Treatment with Brahmi or berry extracts administered individually attenuated selected anxiety-related behaviors, whereas combined treatment did not consistently enhance anxiolytic outcomes beyond single treatments. In contrast, cognitive performance assessed by the novel object recognition test was most consistently improved by the low-dose berry extract, while combination treatments were more clearly associated with histological preservation. Recognition memory-associated parameters were modulated following individual treatments, with the low-dose berry group showing the most consistent effects, while exploratory analyses indicated enhanced novelty-directed exploration in combination-treated groups.

Histopathological findings in the prefrontal cortex and hippocampus supported these behavioral patterns, demonstrating reduced neuronal degeneration and improved tissue organization, particularly in the Brahmi and combination-treated groups.

Overall, these findings indicate domain-specific neuroprotective effects of Brahmi and anthocyanin-rich Thai berry extracts under chronic stress conditions, with individual treatments primarily alleviating anxiety-like behaviors and combined treatment preferentially supporting cognitive and exploratory aspects of behavior. This integrated evidence supports their potential as natural interventions for stress-related anxiety and cognitive modulation.

As for the limitations of this study, this study was conducted exclusively in male rats, precluding assessment of sex-specific responses. Histopathological evaluation was qualitative and based primarily on H&E staining, without quantitative stereological or immunohistochemical analyses. Further studies incorporating quantitative histological and immunohistochemical approaches are warranted to elucidate the underlying cellular and molecular mechanisms. Although the relatively short CUMS duration may have affected the extent of detectable neurodegenerative changes, the behavioral results still showed beneficial effects on anxiety-like behavior and recognition memory-associated parameters. Although combination treatments showed enhanced histological preservation in some brain regions, formal analyses to confirm pharmacological synergy were not performed. Although TAC and HPLC-derived C3G showed a strong overall correlation, deviations may occur at higher concentrations because the spectrophotometric method estimates total monomeric anthocyanins and may be influenced by other absorbing compounds. Therefore, TAC was used primarily for comparative screening, whereas HPLC was used for marker-based quantification. The absence of a standard pharmacological positive control (e.g., fluoxetine or diazepam) may represent a limitation in directly comparing the evaluated extracts with established reference treatments for anxiety-like behaviors. However, the current study design was intended primarily to demonstrate the protective effects of the extracts rather than their therapeutic efficacy. The inclusion of a positive control in future studies would facilitate benchmarking against established pharmacological agents and further support mechanistic interpretation. In addition, biochemical or neuroendocrine stress-related markers, such as corticosterone levels, oxidative stress markers, inflammatory cytokines, synaptic proteins, or neurotransmitter-associated parameters, were not evaluated in the present study. Therefore, confirmation of stress induction was based primarily on established physiological and behavioral alterations observed in the CUMS model.

## Figures and Tables

**Table 1:** Total anthocyanin content, total phenolic content, and antioxidant activities of Thai berry extracts.

Extracts	Bioactive Compounds		Antioxidant Activities		
	TAC (mgC3GE/g)	TPC (mg GAE/g)	DPPH (IC_50_, µg/mL)	ABTS (IC_50_, µg/mL)	FRAP (gFe^2+^/100 gE)
Mulberry	49.98 ± 2.88 ^a^	30.55 ± 0.33 ^a^	163.10 ± 7.50 ^ab^	42.47 ± 5.29 ^a^	15.03 ± 0.50 ^a^
Mamao	14.54 ± 2.88 ^b^	17.95 ± 0.36 ^b^	300.60 ± 37.25 ^a^	133.10 ± 4.26 ^b^	8.21 ± 0.54 ^b^
Ma-kiang	42.97 ± 3.94 ^ac^	50.64 ± 2.48 ^c^	41.48 ± 15.43 ^c^	72.46 ± 9.02 ^c^	28.76 ± 0.72 ^c^
Mixed berry	41.70 ± 1.66 ^c^	32.04 ± 1.78 ^a^	127.75 ± 29.87 ^bc^	50.30 ± 4.50 ^a^	16.36 ± 0.91 ^a^
Trolox	ND	ND	7.41 ± 0.29 ^c^	3.17 ± 0.05 ^d^	42.05 ± 0.34 ᵈ

Values are expressed as mean ± SD (*n* = 3). Within each column, values with different superscript letters are significantly different (*p* < 0.05), whereas values sharing at least one common letter are not significantly different, as determined by one-way ANOVA followed by multiple comparison test. ND, not determined.

**Table 2:** Behavioral validation of the chronic unpredictable mild stress (CUMS) model in control and CUMS-vehicle rats.

Groups	Open-Field Test			Elevated Plus Maze Test		Novel Object Recognition Test		
	Distance (cm)	Time Spent in External Zone (%)	Time Spent in Internal Zone (%)	Distance in Closed Arms (cm)	Number of Entries to Closed Arms (n)	Recognition Index	Time Spent with Novel Object (s)	Number of Entries to Novel Object (n)
Control	1281.00 ± 233.80	96.59 ± 1.07	3.41 ± 1.07	781.30 ± 49.42	4.00 ± 0.42	0.52 ± 0.06	39.31 ± 7.46	8.30 ± 0.93
CUMS	1712.00 ± 225.50	97.01 ± 0.85	2.99 ± 0.85	1012.00 ± 64.42 **	8.25 ± 0.89 ***	0.54 ± 0.05	35.80 ± 6.28	7.25 ± 0.94

Data are presented as mean ± SEM. Statistical significance was determined by unpaired *t*-test. ** *p* < 0.01, *** *p* < 0.001, *n* = 10–12 per group and compared to the control group.

**Table 3:** Chronic unpredictable mild stress procedure sequence.

Day	Stressor	Duration	Day	Stressor	Duration
1	Wet sawdust	20 h	8	Wet sawdust	20 h
2	Food deprivation, light off	18 h, 20 h	9	Food deprivation, light off	18 h, 20 h
3	Remove sawdust	20 h	10	Remove sawdust	20 h
4	Water deprivation, light off	18 h, 20 h	11	Water deprivation, light off	18 h, 20 h
5	Inclined cage 45°	20 h	12	Inclined cage 45°	20 h
6	Forced swimming	10 min	13	Forced swimming	10 min
7	Restraint	1 h	14	Restraint	1 h

## Data Availability

The original contributions presented in this study are included in the article/Supplementary Materials. Further inquiries can be directed to the corresponding authors.

## Supplementary Materials

The following supporting information can be downloaded at https://www.mdpi.com/article/10.3390/ph19070981/s1, Figure S1. Representative HPLC chromatogram of the cyanidin-3-O-glucoside (C3G) standard (40 µg/mL). Chromatographic conditions and system preparation are described in Section 4.4.4; Figure S2. Representative HPLC chromatogram of the standardized anthocyanin-rich mixed Thai berry extract (10 mg/mL). Chromatographic conditions and system preparation are described in Section 4.4.4; Figure S3. Body weight gain (%increase) after 14 days of experimental treatment. Data are presented as mean ± SEM (*n* = 10–12 per group). The control group exhibited significantly greater body weight gain compared with all CUMS-exposed groups (**** *p* < 0.0001). No marked differences in body weight gain were observed among CUMS-exposed treatment groups.
