# Supplementary material for: Protective Effects of Bacopa monnieri Extract, Mixed Thai Berry Extract and Their Combination Against Chronic Unpredictable Mild Stress-Induced Behavioral Changes in Rats
Source: Pharmaceuticals (Basel). 2026 Jun 24;19(7):981. doi: 10.3390/ph19070981 (PMC13414855; doi:10.3390/ph19070981)
Supplement: Supplementary file 1 [file pharmaceuticals-19-00981-s001.zip › pharmaceuticals-4314798-supplementary.pdf]

Supplementary Materials

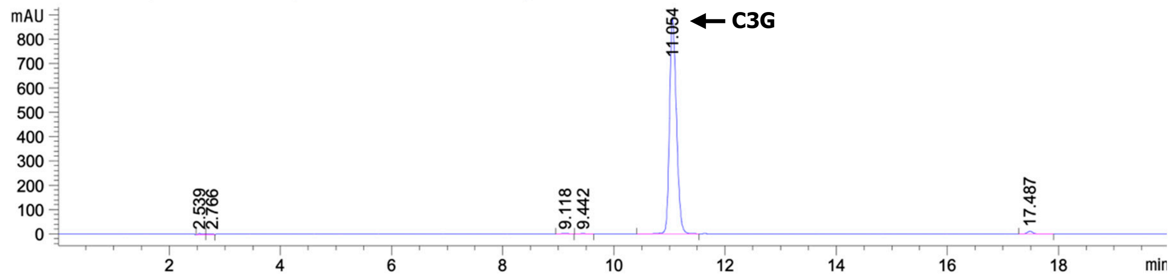

Figure S1. Representative HPLC chromatogram of the cyanidin-3-O-glucoside (C3G) standard (40 µg/mL). Chromatographic conditions and system preparation are described in Section 4.4.4.

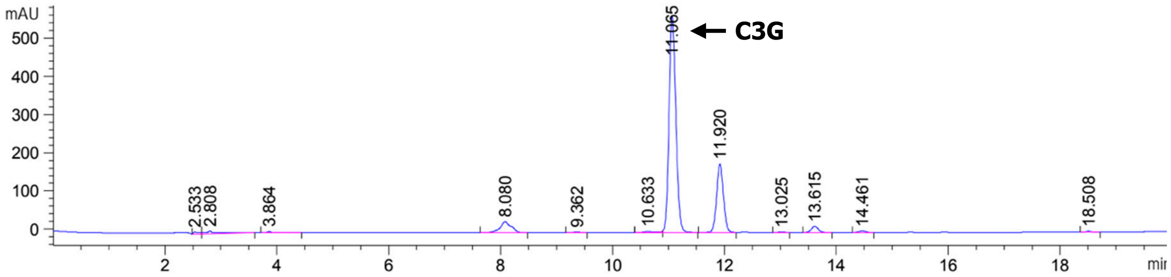

Figure S2. Representative HPLC chromatogram of the standardized anthocyanin-rich mixed Thai berry extract (10 mg/mL). Chromatographic conditions and system preparation are described in Section 4.4.4.

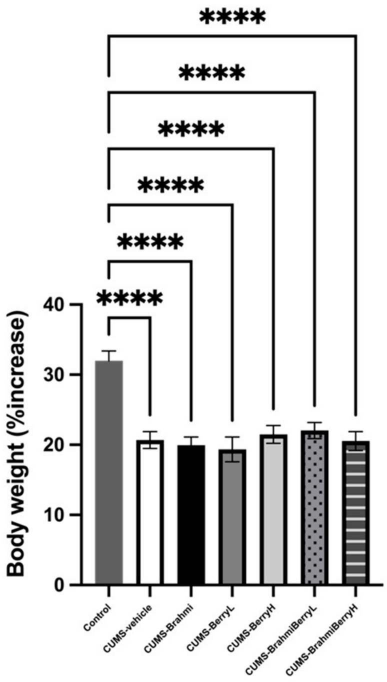

Figure S3. Body weight gain (%increase) after 14 days of experimental treatment. Data are presented as mean ± SEM (n = 10–12 per group). The Control group exhibited significantly greater body weight gain compared with all CUMS-exposed groups (****p < 0.0001). No marked differences in body weight gain were observed among CUMS-exposed treatment groups.

Abbreviations: CUMS, chronic unpredictable mild stress; Brahmi, Brahmi extract-treated group; BerryL, low-dose mixed Thai berry extract-treated group; BerryH, high-dose mixed Thai berry extract-treated group; BrahmiBerryL, Brahmi extract combined with low-dose mixed Thai berry extract-treated group; BrahmiBerryH, Brahmi extract combined with high-dose mixed Thai berry ex-tract-treated group.
